# PREMISE: Posterior Circulation Results Comparing Embolectomy to Medical Intervention in Stroke Emergencies

**DOI:** 10.7759/cureus.6000

**Published:** 2019-10-25

**Authors:** Patrick M Chen, Mohsen Pirastehfar, Lovella Hailey, Melissa Morton, Karen S Rapp, Kunal Agrawal, Royya Modir, Dawn Meyer, Brett C Meyer

**Affiliations:** 1 Neurosciences, University of California San Diego School of Medicine, San Diego, USA

**Keywords:** acute ischemic stroke, acute treatment, basilar artery occlusion, thrombectomy, neurointerventional

## Abstract

Background

Intravenous (IV) tissue plasminogen activator (rt-PA) is a proven therapy for stroke in the acute treatment window. Recent published data has shown efﬁcacy for embolectomy for acute ischemic strokes within up to six, 16 and 24 hours in the anterior circulation but there is no guideline for optimal therapy for patients with posterior circulation stroke, specifically basilar artery occlusion (BAO) outside the standard IV rt-PA treatment window.

Aim

To evaluate differences in outcomes between maximal medical treatment versus thrombectomy in BAO.

Method

We retrospectively evaluated prospectively collected acute stroke code patients from our stroke registry from 7/2004 to 7/2016. Patients who received IV rt-PA were excluded. Patients with evidence of posterior circulation ischemia and a hyper dense artery sign on initial non-contrast CT were included as a surrogate for direct vessel data before 2014. Patients after 9/2014 were selected by evidence of BAO on vessel imaging. All patients were categorized either as endovascular therapy or standard medical treatment alone. Demographics, hospital discharge location and Modified Rankin Scale (mRS) at 90 days were compared. Two-sample t-test and Fisher’s exact test compared continuous and categorical variables across groups respectively.

Results

A total of 18 patients were included (three embolectomy and 15 medical therapy only). There were no significant differences in demographic data (age, gender, race, ethnicity, blood pressure, diabetes mellitus, hypertension, atrial fibrillation, tobacco use, alcohol use and initial NIHSS). Results for outcome and efficacies showed no statistical difference between medical management and endovascular intervention for functional outcome mRS (0-3) at 90 days (p = 0.2) and discharge location of home/inpatient rehabilitation vs other locations (p = 0.52).

Conclusions

Our single-center review showed the expected transition from predominantly medically treated posterior circulation BAOs, to a mixed pattern including embolectomy. Although the sample size was small, this study also illustrates the lack of clear efficacy data for optimal treatment strategies, and the ongoing treatment challenges in posterior circulation stroke population in a population of patients outside the rt-PA window.

## Introduction

Stroke is the fifth leading cause of death and disability in the United States. Posterior circulation stroke accounts for twenty percent of all ischemic strokes [1]. The basilar artery is generally the primary artery that supplies blood flow to the posterior circulation including the brain stem, occipital lobes, and part of the cerebellum and thalami.

A posterior circulation stroke, especially basilar artery occlusion (BAO), has a devastating and extremely high mortality rate (80%) [2-5]. Most of these patients present with alteration of consciousness which often makes the initial diagnosis challenging.

Current evidence based on randomized controlled trial data has resulted in the Food and Drug Administration (FDA) approval for intravenous (IV) recombinant tissue plasminogen activator (rt-PA) within three hours from stroke symptom onset while mechanical embolectomy now has evidentiary support for zero to six hour cases and a specific subset of patient’s out to 24 hours [6-8]. Although IV rt-PA is approved for all arterial stroke distributions, mechanical embolectomy is only strongly supported for anterior circulation large vessel occlusions [8]. There is limited evidence for treatment of stroke with IV rt-PA or mechanical embolectomy in the posterior circulation [9,10]. Posterior circulation stroke trials have met with numerous challenges including difficulty in recruiting sufficient number of patients, patients arriving outside the approved treatment window, or difficulty with determining the diagnosis due to complex presentation of some of these patients.

Considering the potentially devastating consequences of this type of stroke, optimized treatment strategies should be determined. Our aim was to first assess current treatment patterns and relative outcomes for posterior circulation occlusions, as an initial step toward determining future optimal treatment paradigms (medical therapy vs. embolectomy) in posterior circulation large vessel occlusions (LVOs).

## Materials and methods

We retrospectively evaluated prospectively collected acute stroke code patients’ registry data from our center’s institutional review board (IRB) approved datasets; Core C (CC), from July 2004 to September 2014, and Stroke Registry (SR) from October 2014 to July 2016.

Patients who received IV rt-PA were excluded from analyses to assess the effect of medical therapy vs. embolectomy in a clean dataset. Since in our early registry (CC) direct vessel imaging data was not collected, surrogate ascertainment of basilar occlusion was defined by cases with registry documentation of both radiographic evidence of ischemia in the posterior circulation (brainstem/cerebellum/occipital) and the presence of a hyper dense artery sign on initial non-contrast brain CT imaging. This data was combined with the subsequent, more refined, registry (SR) which did include direct documentation of basilar artery occlusion on vessel imaging (CT Angiogram [CTA] or MR Angiogram [MRA]). Patients were then categorized by whether they received endovascular therapy (mechanical embolectomy) or standard medical treatment (antiplatelet or anticoagulant therapy), and the two groups were compared.

Baseline characteristics including age, gender, race, ethnicity, history of diabetes, history of hypertension, blood pressure, history of atrial fibrillation, history of coronary artery disease (CAD), history of smoking or drinking alcohol and initial National institute of Health Stroke Scale (NIHSS) were compared. Functional outcome variables included both hospital discharge location and Modified Rankin Scale (mRS) at 90 days. Good outcomes were defined as hospital location of home or acute rehab, and an mRS of 0 to 3 at 90 days [5]. Safety was assessed by reporting presence of intracranial bleed on brain imaging, after treatment but before discharge. Two-sample t-test and Fisher’s exact test were used to compare continuous and categorical variables across groups respectively. A p-value <0.05 was considered statistically significant.

## Results

A total of 18 stroke code patients with vertebra-basilar occlusion who were outside of standard IV rt-PA consideration were included in our assessment. In our data, 15 received medical therapy while three underwent posterior circulation embolectomy. There were no statistically significant differences in demographic data including age, gender, race, ethnicity, blood pressure, diabetes mellitus, hypertension, atrial fibrillation, tobacco use, alcohol use and initial NIH Stroke Scale Score (NIHSS). Median NIHSS score was not different between groups (p = 0.74) (Table 1).

**Table 1:** Baseline characteristics and comparison between two groups. BP: Blood pressure.

	Embolectomy		
	Negative	Positive	p-value
Age	n = 15	n = 3	0.55
	66.33 (17.17)	71.33 (11.24)	
Gender	n = 15	n = 3	
Female	7 (46.7%)	1 (33.3%)	1
Male	8 (53.3%)	2 (66.7%)	
Race	n = 14	n = 3	
Asian	1 (7.1%)	0 (0%)	0.33
Black	0 (0%)	1 (33.3%)	
White	13 (92.9%)	2 (66.7%)	
Ethnicity	n = 15	n3	
Hispanic	2 (13.3%)	1 (33.3%)	0.442
Not Hispanic	13 (86.7%)	2 (66.7%)	
Atrial Fibrillation	n = 15	n = 3	
No	13 (86.7%)	3 (100%)	1
Yes	2 (13.3%)	0 (0%)	
Systolic BP	n = 15	n = 3	
	150.47 (26)	171 (18)	0.177
Diastolic BP	n = 15	n = 3	
	80.9 (15.3)	102.6 (15.3)	0.11
Hypertension	n = 15	n = 3	
No	3 (20%)	1 (33.3%)	1
Yes	12 (80%)	2 (66.7%)	
Diabetes	n = 15	n = 3	0.522
No	10 (66.7%)	3 (100%)	
Yes	5 (33.3%)	0 (0%)	
Tobacco Use	n = 15	n = 3	1
Other	14 (93.3%)	3 (100%)	
Present	1 (6.7%)	0 (0%)	
Alcohol Use	n = 15	n = 3	1
Other	13 (86.7%)	3 (100%)	
Present	2 (13.3%)	0 (0%)	
Initial NIH Stroke Scale	n = 15	n = 3	0.74
	13 (8)	17.3 (19.66)	

There were no intracranial hemorrhages noted in either treatment group. For patients with 90-day outcome assessments (a variable which required patient consent to obtain for the registry), good outcome was not different for mRS (p = 0.20) (Table 2).

**Table 2:** Ninety days Modified Rankin Scale (mRS) outcome and comparison between two groups.

		Modified Rankin Scale (mRS)
	mRS >3 (n = 1)	mRS ≤ 3 (n = 4)
Embolectomy		
Negative	0 (0%)	4 (100%)
Positive	1 (100%)	0 (0%)

There was no difference in discharge location between groups (p = 0.52) (Table 3).

**Table 3:** Discharge location of home/acute rehabilitation versus death/hospice/skilled nursing facility.

		Discharge Location	
	Death/Hospice/Skilled Nursing Facility (n = 13)	Home/Acute Rehab (n = 5)	p-value
Embolectomy			
Negative	10 (76.9%)	5 (100%)	0.522
Positive	3 (23.1%)	0 (0%)	

## Discussion

This exploratory assessment sought to determine current treatment strategies for posterior circulation occlusions (BAOs) outside of the standard IV rt-PA window, prior to a more prospective assessment of standardized treatment options.

Our single center experience showed the expected transition from predominantly medically treated posterior circulation BAOs, to a more mixed pattern including embolectomy over time. This is in keeping with the evolution of evidence from landmark trials supporting the use of embolectomy for LVOs. The fact that the majority of patients were treated medically overall is consistent with the lack of evidence for embolectomy before the publication of multiple positive embolectomy trials since 2014 [6-8,11]. Although the data is strongest for the anterior circulation embolectomy, this therapy is being applied to LVOs at many centers for lesions in other vascular distributions as well.

We did not find a difference in 90-day outcomes for either modality of treatment. This is likely at least partially due to small sample size, and a future, larger study would also utilize multivariate analysis. Nevertheless this study does illustrate the lack of clear efficacy data for optimal treatment strategies in BAOs or posterior circulation LVOs, and the ongoing treatment challenges in these populations.

Our choice to exclude IV rt-PA patients may have lessened the sample size further. Nevertheless, it provided a clean study population and helped assess our goal of determining best therapy for patients outside of the standard IV rt-PA treatment window, a common clinical situation. A recent small retrospective demonstrated possible improved outcomes in BAOs that required delayed thrombectomy [12]. Other national databases are being sought to assess the idea of conservative management versus thrombectomy of patients outside the TPA window in a larger data set.

There is great need in the literature to determine the current environment of treatment options and relative outcome. The current literature suffers from small numbers of patients being reported, in part because of the devastating nature of the disease and difficulty in enrollment into these types of trials. The largest prospective cohort for BAO (BASIC Study) evaluated different treatment intervals within the modality, but time to treatment was not recorded for medical therapy and this modality was not compared to thrombectomy [2]. A recent large meta-analysis comparing IV r-tPA to intra-arterial therapy, found subgroup superiority for thrombectomy with intra-arterial therapy, but did not address our cohort of patient [13]. There is growing interest in thrombectomy selection factors (imaging findings, low NIHSS) that may predict better outcome post thrombectomy in this clinically challenging cohort of patients [14].

Ultimately, at this time, there is limited evidence to strongly support aggressive treatment strategy in posterior circulation LVO patients such as BAOs outside the rt-PA window, and further prospective, large trial assessments are warranted.

## Conclusions

Our data was able to assess current treatment strategies and adds to the overall limited data set on posterior LVOs outside of the rt-PA window. We did not find a difference in outcomes related to modality studied, with the caveat of small sample size and limited power. Our present analysis illustrates the ongoing treatment challenges in posterior circulation LVOs in general and specifically in BAOs. There continues to be a need for further investigation in larger sample sizes, and in the setting of prospective randomized clinical trials.
